# Long-Term *In Vitro* Culture of the Syphilis Spirochete *Treponema pallidum* subsp. *pallidum*

**DOI:** 10.1128/mBio.01153-18

**Published:** 2018-06-26

**Authors:** Diane G. Edmondson, Bo Hu, Steven J. Norris

**Affiliations:** a Department of Pathology and Laboratory Medicine, McGovern Medical School, University of Texas Health Science Center at Houston, Houston, Texas, USA; b Department of Microbiology and Molecular Genetics, McGovern Medical School, University of Texas Health Science Center at Houston, Houston, Texas, USA; NIAID, NIH

**Keywords:** *Treponema pallidum*, cell culture, cell structure, electron microscopy, infectivity, physiology, spirochetes

## Abstract

Investigation of Treponema pallidum subsp. pallidum, the spirochete that causes syphilis, has been hindered by an inability to culture the organism continuously *in vitro* despite more than a century of effort. In this study, long-term logarithmic multiplication of T. pallidum was attained through subculture every 6 to 7 days and periodic feeding using a modified medium (T. pallidum culture medium 2 [TpCM-2]) with a previously described microaerobic, rabbit epithelial cell coincubation system. Currently, cultures have maintained continuous growth for over 6 months with full retention of viability as measured by motility and rabbit infectivity. This system has been applied successfully to the well-studied Nichols strain of T. pallidum, as well as to two recent syphilis isolates, UW231B and UW249B. Light microscopy and cryo-electron microscopy showed that *in vitro*-cultured T. pallidum retains wild-type morphology. Further refinement of this long-term subculture system is expected to facilitate study of the physiological, genetic, pathological, immunologic, and antimicrobial susceptibility properties of T. pallidum subsp. pallidum and closely related pathogenic *Treponema* species and subspecies.

## IMPORTANCE

Syphilis, a sexually transmitted disease with a global distribution, is caused by a spiral-shaped bacterium called Treponema pallidum subspecies pallidum. Previously, T. pallidum was one of the few major bacterial pathogens that had not been cultured long-term *in vitro* (in a test tube), greatly hindering efforts to better understand this organism and the disease that it causes. In this article, we report the successful long-term cultivation of T. pallidum in a tissue culture system, a finding that is likely to enhance our ability to obtain new information applicable to the diagnosis, treatment, and prevention of syphilis.

## INTRODUCTION

Syphilis is a multistage sexually transmitted infection of worldwide importance, with estimates of total disease burden ranging from 18 to 56 million individuals (1–4). Treponema pallidum subsp. pallidum (T. pallidum), the causative agent of syphilis, was first identified by Schaudinn and Hoffman in 1905 (5, 6) as “very light, thin spiraled microorganisms, turning around their largest length and moving back and forth.” Rapid progress in the study of this bacterium was made within 5 years, with the verification of the presence of spirochetes in experimentally infected animals by Metchnikoff and Roux (7), the invention of dark-field microscopy for easy visualization of T. pallidum by Karl Landsteiner, development of the first serological test for syphilis by von Wassermann et al. (8), and the introduction of arsphenamine as an effective, relatively nontoxic antisyphilis agent by Paul Ehrlich (9, 10). Successful culture of T. pallidum was reported almost immediately and during the subsequent decades (11, 12), but these reports were found to be either irreproducible or the result of contamination with nonpathogenic *Treponema* species that colonize human skin (13). During the 1970s, progress was made in characterizing some T. pallidum physiological properties, most notably its microaerophilic nature and improved survival in the presence of mammalian cells (14).

In 1981, Fieldsteel, Cox, and Moeckli (15) reported the consistent occurrence of up to 100-fold multiplication of T. pallidum in a coculture system consisting of Sf1Ep cottontail rabbit epithelial cells, a modified tissue culture medium with heat-inactivated fetal bovine serum, dithiothreitol (DTT) as a reducing agent, and a microaerobic atmosphere containing 1.5% O_2_. These results were reproduced in hundreds of experiments (primarily by the Cox and Norris groups) and reported in over 25 publications (reviewed in references 14 and 16). However, treponemal multiplication and survival were limited to 12 to 18 days, despite efforts to refine this system. Attempts to subculture T. pallidum provided little improvement in the cumulative fold increase or survival of the bacterium (14, 16, 17). The same limitation has existed for the closely related organisms that cause yaws (T. pallidum subsp. pertenue), bejel (T. pallidum subsp. endemicum), pinta (Treponema carateum), and venereal spirochetosis in rabbits and hares (T. paraluiscuniculi) (18, 19). The inability to culture these organisms continuously *in vitro* has necessitated their propagation in rabbits for use in research, greatly hindering investigation of these important pathogens.

In this study, we utilized a modification of the method described by Fieldsteel et al. (15, 16) to achieve reproducible, long-term multiplication of T. pallidum subsp. pallidum in a tissue culture system. In ongoing experiments, T. pallidum multiplication and full viability have been maintained for up to 27 passages over a period of 6 months. Results obtained with the Nichols reference strain have been verified using two recent T. pallidum isolates. The *in vitro*-cultured treponemes retained their characteristic ultrastructure (as determined by cryoelectron microscopy) and full infectivity (as demonstrated by rabbit inoculation experiments). The availability of this culture system is likely to lead to a better understanding of T. pallidum physiology, structure, gene expression, regulatory pathways, pathogenesis, immunologic properties, and antimicrobial susceptibility.

## RESULTS

### Long-term culture of T. pallidum*.*

T. pallidum subsp. pallidum Nichols, isolated from the cerebrospinal fluid of a neurosyphilis patient in 1912 (20) and the reference strain for this subspecies, was utilized for most experiments, whereas strains UW231B and UW249B, isolated from the blood of untreated syphilis patients in Seattle, WA, were used to examine the applicability of the culture method to other syphilis strains. These and (to our knowledge) all other available T. pallidum strains were isolated and propagated by inoculation of rabbits. The *in vitro* cultures were initiated with frozen preparations of T. pallidum previously extracted from infected rabbit testes and stored at −80°C.

In preliminary studies, multiple modifications of the T. pallidum cultivation medium (TpCM) described by Cox (16) were examined to determine their effects on treponemal survival and growth. The basal medium of TpCM consists of Eagle’s minimal essential medium (MEM) with added nonessential amino acids. We found that replacement of the Eagle’s MEM component with the more complex CMRL 1066 tissue culture medium (21) resulted in increased yields and improved retention of motility of T. pallidum during primary culture *in vitro*. The CoCl_2_, cocarboxylase, catalase, and bovine superoxide dismutase components of TpCM did not have a measurable effect on T. pallidum *in vitro* in our analysis and thus were omitted from our formulation. We have named the modified medium TpCM-2 to reflect its similarity to the medium described by Cox (16).

A pilot experiment provided a positive indication that prolonged *in vitro* multiplication of T. pallidum may be possible with TpCM-2 and Sf1Ep cottontail rabbit epithelial cells. In this study, two different series of subcultures were initiated from a primary T. pallidum Nichols culture: one with sequential subcultures on days 8, 16, and 28, and the other on days 18 and 26. Significant multiplication occurred in each of the subcultures, except for the last transfer in the series consisting of days 8, 16, and 28 (see Fig. S1 in the supplemental material). In addition, motility was retained at above 50% until 28 days. Cumulative fold increase (the product of the fold increase values for each subculture) provided a measure of the sustained multiplication under these conditions (Fig. S1C). While the primary culture increase reached a maximum of 57-fold, the two subculture series achieved cumulative fold increases of 978 and 1,105, respectively. Motility in the subcultures was lost by day 36. We therefore reasoned that prolonged survival and growth of T. pallidum may be obtained by shortening the subculture interval and thus maintaining conditions that are more homeostatic.

10.1128/mBio.01153-18.1 FIG S1  Pilot experiment showing continued multiplication of T. pallidum subsp. pallidum Nichols in subcultures during coincubation with Sf1Ep cells in TpCM-2 medium. Primary cultures were inoculated with a frozen preparation of rabbit-propagated T. pallidum. Cultures were trypsinized on days 8, 16, 28, and 36 for T. pallidum concentration and motility determinations. One set of subcultures were performed sequentially on days 8, 16, and 28; subcultures were done on days 16 and 28 in the second set. Cumulative 978-fold and 1,105-fold growth increases were observed in the two subculture series. (A) T. pallidum organisms per culture. (B) Percent motility. (C) Cumulative fold increase. T. pallidum per-culture and percent motility values represent means + SE of results from triplicate cultures. Download FIG S1, TIF file, 4.5 MB. Copyright © 2018 Edmondson et al. 2018 Edmondson et al. This content is distributed under the terms of the Creative Commons Attribution 4.0 International license.

The current culture procedure (outlined in Table 1) utilizes coincubation of T. pallidum with Sf1Ep cells in TpCM-2 at 34°C in a microaerobic atmosphere consisting of 1.5% O_2_ and 5% CO_2_, with the balance consisting of N_2_. Under these conditions, ~90% of the treponemes adhere to the surface of the Sf1Ep cells and multiply by binary fission. T. pallidum organisms were dissociated from the Sf1Ep cells by treatment with trypsin and EDTA, and the resulting suspension was used for quantitation by dark-field microscopy and the transfer of organisms to fresh subcultures. In some experiments, a portion of the medium was replaced with fresh TpCM-2 at 3 to 4 days. The typical subculture interval was 6 to 7 days, although this period could be prolonged if the T. pallidum concentration was low. Triplicate cultures in 6-well cluster dishes were utilized for each time point with each culture being used to inoculate a separate subculture, thus maintaining three parallel biological replicates. Details regarding the methodologies utilized are described in Materials and Methods.

**TABLE 1:** Outline of T. pallidum long-term-cultivation parameters

Parameter
Six-well cluster dishes (9 cm^2^/well)
No. of Sf1Ep cells/well, 1 × 10^5^
Vol of TpCM-2 medium, 2 to 4 ml
Preincubation of medium and Sf1Ep cells in an atmosphere of 1.5% O_2_ and 5% CO_2_, with N_2_ making up the balance
Inoculation with 0.5–1.25 × 10^6^ T. pallidum organisms per well, using triplicate wells per condition
Incubation for 6–7 days at 34°C in an atmosphere of 1.5% O_2_ and 5% CO_2_, with N_2_ making up the balance
Replacement of 50% of medium after 3–4 days (optional)
Trypsin/EDTA treatment of “donor” culture
Inoculation of “recipient” culture with 0.1 ml–0.5 ml of trypsinized “donor” culture
Subculture at 6–7-day intervals

**FIG 1:**
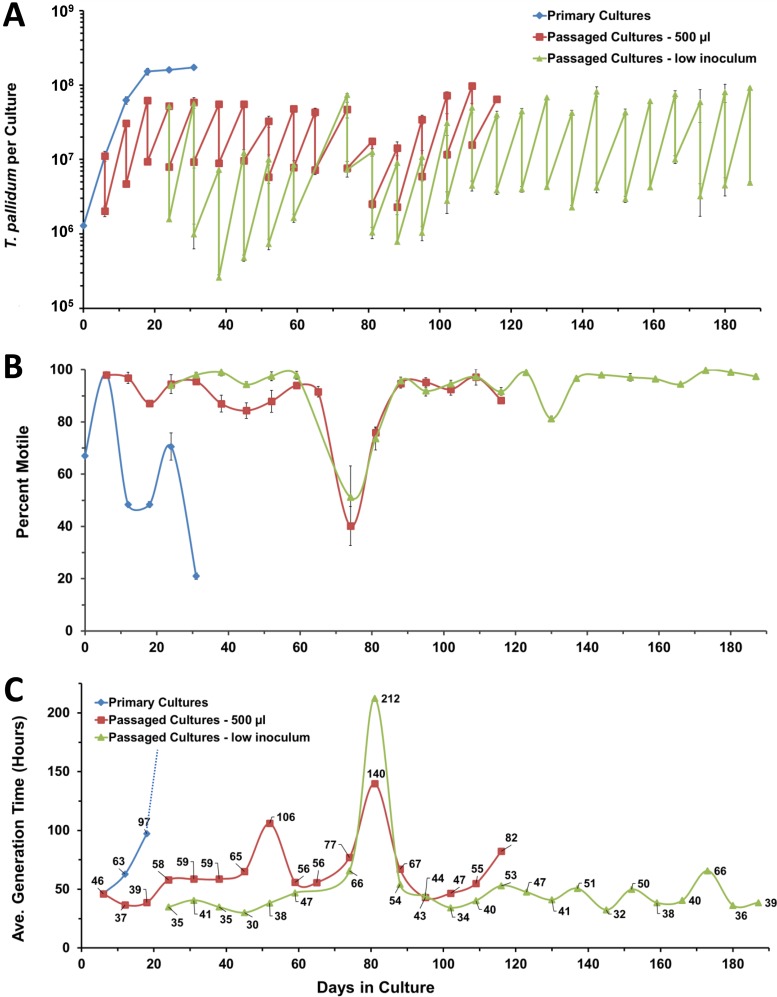
Long-term cultivation of T. pallidum subsp. pallidum Nichols in a tissue culture system (experiment 1). Primary cultures represent the results obtained without subculture. The 500-µl passages used transfer of that volume for each subculture, whereas the low-inoculum passages used lower volumes (100 µl to 250 µl). (A) In this “sawtooth” plot, the number of T. pallidum organisms per culture upon harvest and the number inoculated in the next subculture are shown for each time point. (B) Percent motility provides a measure of viability. (C) Average (Ave.) generation time, representing the time in hours divided by log_2_(fold increase) for each time point. T. pallidum per-culture and percent motility values represent means ± SE of results from three biological replicates.

Using these conditions, all five of the T. pallidum cultivation experiments that we have undertaken to date have exhibited consistent, long-term multiplication with retention of motility (Table 2); four of these cultures are still ongoing. The current results of our longest-ongoing T. pallidum culture experiment (experiment 1 [Exp. 1]) are depicted in Fig. 1. The numbers of T. pallidum organisms per culture are shown in the “sawtooth” graph in Fig. 1A, in which the mean numbers (± standard errors [SE]) of organisms harvested and inoculated in the subcultures are shown for each time point. In this and subsequent experiments, parallel nonpassaged (“primary”) cultures were included for comparison. During the first three subcultures, a volume of 0.5 ml (labeled “500 µl,” from the total trypsinized culture volume of ~2.4 ml) was used. However, we noted that these cultures reached a maximum number of roughly 5 × 10^7^ T. pallidum organisms per culture and exhibited some loss of motility. Therefore, beginning on day 24, parallel subcultures were begun that contained a lower inoculum of 0.1 to 0.25 ml (“low inoculum”). The transferred volume was typically 0.25 ml for these cultures but was adjusted to a lower volume if a particularly high T. pallidum concentration was observed during a brief microscopic examination. Note that a given volume of trypsinized culture was used rather than adjustment of the inoculum to a certain number of bacteria (which would require an accurate microscopic concentration determination) to minimize the transfer time and thus the exposure to atmospheric levels of oxygen. A typical transfer time was 20 to 30 min.

**TABLE 2:** Summary of T. pallidum subculture experiments

Experiment	Status	Strain	No. of days in culture	Passage no.	Cumulative fold increase	No. of generations	Avg generation time (h)^a^	Minimum generation time (h)^b^
1	Ongoing	Nichols	187	26	1.1 ×10^30^	100	45	33
2	Ongoing	Nichols	189	27	2.4 ×10^31^	104	44	33
3	Ongoing	Nichols^c^	119	17	2.4 ×10^19^	64	43	36
4	Ongoing	UW231B	70	9	2.6 × 10^9^	31	55	44
5	Complete	UW249B^d^	40	6	6.5 × 10^7^	26	54.6	46

a Avg generation time = (time in hours)/(number of generations) for all days in culture, excluding the first culture period.

b Minimum generation time = average of the 5 lowest subculture generation times (3 for experiment 5).

c Experiment 3 was initiated from frozen stocks of a day 54 culture from experiment 1. All other experiments were initiated from frozen preparations of rabbit-propagated T. pallidum.

d Experiment 5 was discontinued after day 40 because of fungal contamination.

In this representative experiment, the primary cultures multiplied exponentially for the first 12 days, at which time growth slowed and motility decreased (Fig. 1). In contrast, continued multiplication of T. pallidum occurred in each of the subsequent 25 subcultures, indicating that the replicative potential of the treponemes was retained. In addition, motility (as a measure of cellular viability) was generally maintained at a high level, whereas the motility of the primary cultures decreased dramatically on day 12 (Fig. 1B). A decrease in motility did occur in the subcultures on day 74, when the subculture interval was inadvertently extended to 9 days. However, viability recovered during the following two subcultures, indicating the outgrowth of a subset of organisms that had not been irreversibly damaged. The reduction in the percentage of motile organisms correlated with an increase in the average generation time over the 7-day period of the subsequent subculture (Fig. 1C). Thereafter, the growth of T. pallidum recovered to the prior rate. At the time of writing, this experiment had been ongoing for 187 days, or over 6 months.

**FIG 2:**
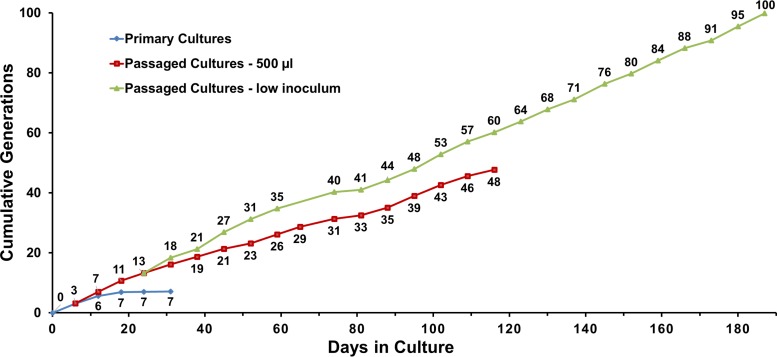
Cumulative generations during *in vitro* culture of T. pallidum subsp. pallidum Nichols in experiment 1. Increases in the primary cultures occurred only within the first 12 days. The generations accumulated more rapidly in the low-inoculum cultures than in the 500-µl inoculum cultures until the latter were discontinued on day 116.

The value corresponding to the cumulative generations for the high-inoculum (500-µl) cultures lagged behind that obtained with the parallel low-inoculum cultures (Fig. 2). Excluding the variant subcultures on days 74 and 80, the average generation time ranged from 37 to 106 h for 0.5-ml-inoculum cultures (mean ± standard deviation [SD] = 63 ± 18 h) and from 30 to 66 h for the low-inoculum cultures (41 ± 8 h) for the time points shared by the two subculture series. A *t* test comparison showed that the generation time was significantly lower (*P* ≤ 0.05) in the subcultures receiving a lower inoculum, most likely because optimal multiplication was sustained for a longer time due to slower depletion of required nutrients or accumulation of toxic compounds. For this reason, the high-inoculum portion of this experiment was discontinued on day 116.

Very similar results were obtained in a replicate experiment (Exp. 2), which is ongoing at 189 days of culture and 27 subcultures (Fig. S2). Multiplication and motility have been consistently maintained except for a downturn on days 98 to 105 for unknown reasons, with a subsequent recovery. Overall, this experiment has shown a cumulative fold increase of 2.4 ×10^31^, or 104 generations.

10.1128/mBio.01153-18.2 FIG S2  *In vitro* culture of T. pallidum subsp. pallidum Nichols (Exp. 2). Parameters are as described in Fig. 1. (A) Number of T. pallidum organisms per culture. (B) Percent motility. (C) Average generation time. T. pallidum per-culture and percent motility values represent means ± SE of results from triplicate cultures. Download FIG S2, TIF file, 7.4 MB. Copyright © 2018 Edmondson et al. 2018 Edmondson et al. This content is distributed under the terms of the Creative Commons Attribution 4.0 International license.

### Requirement for mammalian cells.

**FIG 3:**
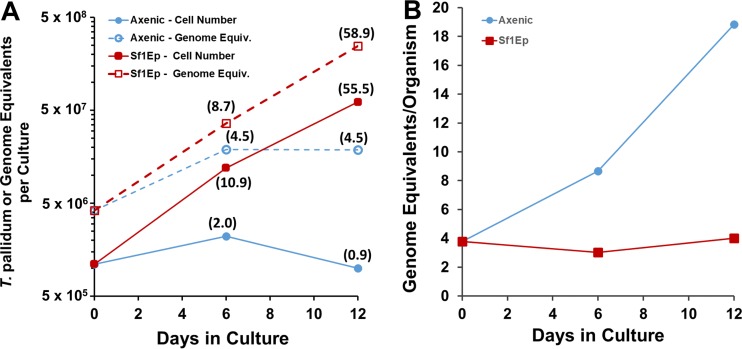
Multiplication and genome replication of T. pallidum Nichols in Sf1Ep cocultures and axenic cultures. Fold increases are shown in parentheses. The axenic cultures were initiated in parallel with the Sf1Ep cocultures in experiment 1 (Table 2) (Fig. 1), using the same frozen stock of T. pallidum. (A) The number of T. pallidum organisms per culture increased only ~2-fold in axenic cultures but increased over 55-fold for cocultures with Sf1Ep cells. Genome equivalent (Equiv.) increases were similar to bacterial cell number increases for the Sf1Ep cocultures but were somewhat higher than the cell number increases for the axenic cultures. (B) Numbers of genome Equiv. per cell remained stable for the exponentially growing cocultures but increased for the axenic cultures. T. pallidum per-culture and genome equivalent values represent means ± SE of results from triplicate cultures.

Experiments were performed to examine whether TpCM-2 medium alone could support the long-term survival and growth of T. pallidum Nichols in the absence of mammalian cells (i.e., in axenic cultures). Quantitative PCR was also used to determine the number of T. pallidum genome equivalents per culture during the course of these studies. The results of two experiments (Fig. 3; see also Fig. S3) were similar and showed that small (2.0-fold and 2.2-fold) but statistically significant (*P* values of 0.013 and 0.039) increases in the number of T. pallidum organisms occurred in axenic cultures during the first 6 days of culture. Motility was also maintained at high levels (99% and 95%) at this time point. However, the T. pallidum yield was not significantly higher than the inoculum on day 12 of culture, and motility had decreased (43% and 83%). Interestingly, the number of genome equivalents per cell increased dramatically in the axenic T. pallidum population, while remaining relatively stable in the actively multiplying T. pallidum population in the Sf1Ep cocultures. Thus, only modest multiplication (roughly 1 cell division) occurred in the axenic cultures in these experiments, but this limitation was apparently not due to the lack of genomic replication.

**FIG 4:**
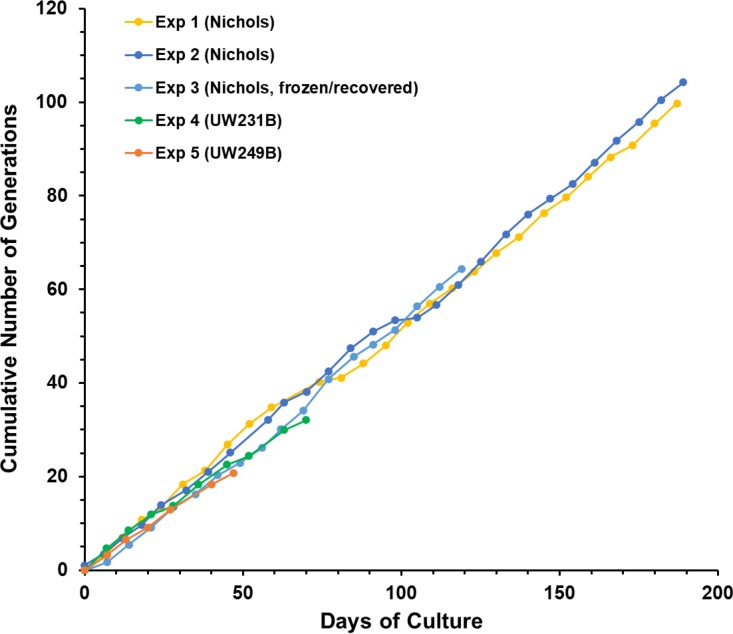
Consistency of T. pallidum multiplication *in vitro*, as illustrated by cumulative generation values from experiments 1 to 5.

10.1128/mBio.01153-18.3 FIG S3  Replication of the experiment in Fig. 4, showing the effects of Sf1Ep coculture and axenic culture on T. pallidum cell numbers and genome equivalents during *in vitro* incubation. Download FIG S3, TIF file, 5 MB. Copyright © 2018 Edmondson et al. 2018 Edmondson et al. This content is distributed under the terms of the Creative Commons Attribution 4.0 International license.

### Successful freezing and recovery of *in vitro*-cultured T. pallidum*.*

*In vitro*-cultured T. pallidum Nichols organisms from a subculture harvested on day 54 of Exp. 1 were frozen at −80°C in TpCM-2 with 15% glycerol. The organisms were thawed 25 days later and used to inoculate fresh cultures (Exp. 3). The thawed T. pallidum exhibited only a 3.1-fold increase during the first 7-day culture period but consistently showed a high replication rate thereafter (e.g., a 14.1-fold increase, or 3.8 doublings, during the second 7-day period). This culture has been passaged continuously for 119 days (17 subcultures) and has undergone a 2.4 ×10^19^-fold cumulative fold increase or 26 generations (Table 2; see also Fig. S4). Thus, *in vitro*-cultured T. pallidum can be successfully frozen and recovered, raising the possibility of continuous *in vitro* culture of T. pallidum without a need for rabbit inoculation.

10.1128/mBio.01153-18.4 FIG S4  Successful revival and culture of T. pallidum subsp. pallidum Nichols from a frozen stock of *in vitro*-cultured organisms. These data are from experiment 3 (see Table 2). A portion of a trypsin-dissociated day 54 culture from experiment 1 (Table 2) was supplemented with 15% (vol/vol) glycerol and frozen at −80°C. The sample was thawed 25 days later and used to inoculate a new set of Sf1Ep cultures. Thawed T. pallidum was treated as a primary culture (without subculture) or was subcultured at 6-to-7-day intervals (with subculture). On day 35, the medium volume was changed from 2 ml to 4 ml. Separate cultures inoculated with frozen, rabbit-propagated T. pallidum were included as controls. (A) Number of T. pallidum organisms per culture. (B) Percent motility. (C) Average generation time per culture period. Download FIG S4, TIF file, 9.6 MB. Copyright © 2018 Edmondson et al. 2018 Edmondson et al. This content is distributed under the terms of the Creative Commons Attribution 4.0 International license.

### Culture of additional T. pallidum strains.

To determine whether this *in vitro* culture technique can be applied to other T. pallidum strains, we performed long-term culture experiments with two recent syphilis isolates, UW231B and UW249B. These strains are members of the so-called SS14 clade of T. pallidum subsp. pallidum that is currently predominant in the United States, Europe, and many other regions (22–24). As with the prior studies with the Nichols strain, cultures were inoculated with frozen preparations of the UW231B and UW249B strains prepared from infected rabbit testes. The *in vitro* cultures of UW231B and UW249B exhibited long-term multiplication with retention of motility and wild-type morphology. The UW231B cultures are ongoing at 70 days of culture with 9 passages, with a cumulative fold increase of 2.6 × 10^9^ (31 generations) (Table 2; see also Fig. S5). Similar results were obtained with UW249B (Table 2; see also Fig. S6), but this culture series was discontinued on day 46 due to subsequent fungal contamination.

10.1128/mBio.01153-18.5 FIG S5  Long-term *in vitro* culture of T. pallidum subsp. pallidum UW231B (experiment 4). Sf1Ep cultures were inoculated with frozen, rabbit-derived UW231B. The cultures were either incubated without subculture but with partial medium replacement every 6 to 7 days (primary cultures) or subcultured at 6-to-7-day intervals. (A) Number of T. pallidum organisms per culture. (B) Percent motility. (C) Average generation time. T. pallidum per-culture and percent motility values represent means ± SE of results from triplicate cultures. Download FIG S5, TIF file, 6 MB. Copyright © 2018 Edmondson et al. 2018 Edmondson et al. This content is distributed under the terms of the Creative Commons Attribution 4.0 International license.

10.1128/mBio.01153-18.6 FIG S6  Long-term *in vitro* culture of T. pallidum subsp. pallidum UW249B (experiment 5). Conditions were as described for UW231B in Fig. S4. The experiment was discontinued at day 46 due to fungal contamination in the subsequent subculture. (A) Number of T. pallidum organisms per culture. (B) Percent motility. (C) Average generation time. T. pallidum per-culture and percent motility values represent means ± SE of results from triplicate cultures. Download FIG S6, PDF file, 0.1 MB. Copyright © 2018 Edmondson et al. 2018 Edmondson et al. This content is distributed under the terms of the Creative Commons Attribution 4.0 International license.

### Consistency of *in vitro* culture.

The growth curves in cumulative generations of the five long-term cultures listed in Table 2 are provided in Fig. 4. The results obtained were remarkably consistent, exhibiting very similar growth rates over time. At the time of this writing, two cultures (Exp. 1 and Exp. 2) have now been maintained for over 6 months (187 and 189 days, respectively). Additionally, over 20 “side” experiments have been performed with these long-term cultures and yielded similar results in terms of retention of viability and multiplication. Up to 2.4 ×10^31^-fold increases, or 104 generations (cell divisions), have been observed in serially passaged cultures to date (Table 2), with no apparent change in growth rates or reduction in motility (as a measure of viability) over time. By comparison, growth in nonpassaged (primary) cultures was limited to 134- to 163-fold increases (7.2 to 7.3 generations), with loss of motility and decreases in cell numbers after 20 to 31 days.

The doubling time of a bacterium is defined as the time between cell divisions during optimal growth, i.e., the mid-log phase of exponential growth. Magnuson et al. (25) determined the doubling time of T. pallidum during rabbit infection to be 30 h using the relationship between dosage and the time of dermal lesion development, whereas Cumberland and Turner (26) calculated a remarkably similar value of 33 h based on extraction and enumeration of organisms at different time points following intratesticular inoculation of rabbits. In our *in vitro* cultures, we have thus far determined cell numbers only at the beginning and end of 6-to-7-day subculture intervals, likely including the lag, exponential, and early stationary phases. Therefore, we can calculate only the average generation time for each subculture, which could be expected to be higher than the exponential doubling time. The average generation times over the entire course of experiments 1 to 5 ranged from 44 to 52 h (Table 2). However, as noted previously, some subcultures had markedly higher generation times (see, e.g., Fig. 1C). To estimate the minimum generation time, the lowest five subculture generation time values were averaged for each experiment (Table 2); only the lowest 3 generation times were included for Exp. 5, because the experiment had only six time points. For the Nichols strain (experiments 1, 2, and 3), the minimum generation time values were 33.3, 33.3, and 35.6 h, respectively, whereas somewhat higher values were obtained with strains UW231B (43.8 h) and UW249B (45.8 h). The results for UW231B and UW249B are based on single experiments; therefore, we do not know at this point if this dissimilarity is due to experimental variation or to true biological differences in growth rates. Nevertheless, the results obtained to date indicate that the estimated multiplication rate of T. pallidum *in vitro* is quite similar to that which occurs during experimental rabbit infection.

### Infectivity.

**FIG 5:**
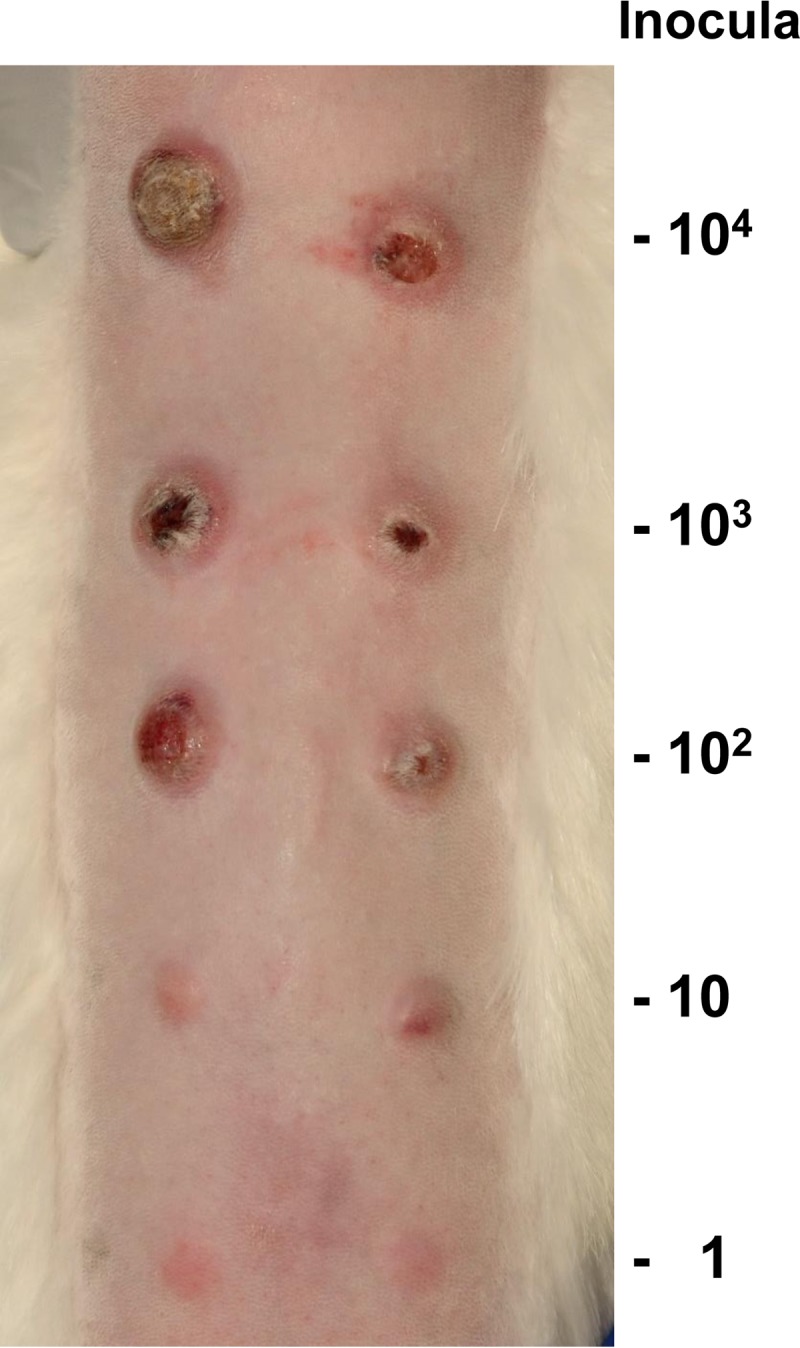
T. pallidum subsp. pallidum Nichols organisms cultured *in vitro* for 116 days were fully infectious in the rabbit intradermal infection model. This photograph of one of the three rabbits in infectivity experiment A (Table 3) was taken on day 45 postinoculation. Paired sites were inoculated intradermally with the dosages shown. Sites inoculated with higher doses had undergone ulceration, as is typical of intradermal infections in rabbits.

To determine whether T. pallidum remained infectious during *in vitro* culture, two experiments (A and B) were performed in which serial dilutions of T. pallidum Nichols from 45- and 116-day *in vitro* cultures were inoculated intradermally into the shaved backs of rabbits, and the injection sites were observed daily for the development of lesions. Under these conditions, the time of lesion development is inversely proportional to the number of infectious organisms injected, i.e., higher dosages produce lesions more rapidly (25, 27, 28). The results demonstrated that the *in vitro*-cultured T. pallidum remained fully infectious, with dilutions calculated to contain as few as one organism yielding lesions (Fig. 5) (Table 3). The times of lesion development obtained in a prior study with freshly extracted, rabbit-derived T. pallidum Nichols were 6.2, 8.9, 14.8, and 17.2 days for inocula of 10^5^, 10^4^, 10^3^, and 2 × 10^2^, respectively (28); the values obtained in the experiments reported here were comparable. In both experiments, sites injected with as few as 100 organisms developed lesions at all sites (Table 3). In experiment A (day 45 organisms), 2 of 4 sites inoculated with 10 T. pallidum were positive; in experiment B (day 116 bacteria), all sites injected with 10 organisms were positive and 3 of 6 sites were positive at the one-organism injection sites. Thus, the 50% infective dose (ID_50_) of *in vitro*-cultured T. pallidum was 10 organisms in experiment A and was 1 organism in experiment B and can be considered to be ≤10 organisms. Needle aspirates from representative lesions at each dosage were consistently positive for motile treponemes by dark-field microscopy. Exceptions were the lesions from the lowest positive dosages in the two experiments, most likely because the immune response against the previously developing lesions limited the concentration of T. pallidum and the lesion size at the lower-dose sites (Fig. 5). Overall, these results demonstrate that T. pallidum organisms cultured *in vitro* in our studies retain their ability to multiply and to cause disease in a widely used animal model of syphilitic infection.

**TABLE 3:** Retention of infectivity by *in vitro*-cultured T. pallidum Nichols

T. pallidum dosage per site	No. of lesions/ no. of sites inoculated	Day of lesion development	Avg day of lesion development
Experiment A: inoculum = 45-day culture			
460,000	4/4	7, 7, 7, 7	7.0
100,000	4/4	8, 8, 8, 8	8.0
10,000	4/4	9, 11, 11, 11	10.5
100	3/4	18, 19, 20	19.0
10	2/4	21, 26	23.5
Experiment B: inoculum = 116-day culture			
10,000	6/6	11, 11, 11, 11, 11, 12	11.2
1,000	6/6	11, 11, 11, 11, 11, 12	11.2
100	6/6	14, 16, 16, 17, 17, 18	16.3
10	6/6	23, 24, 24, 25, 29, 29	25.7
1	3/6	19, 36, 36	30.3

### Bacterial ultrastructure.

**FIG 6:**
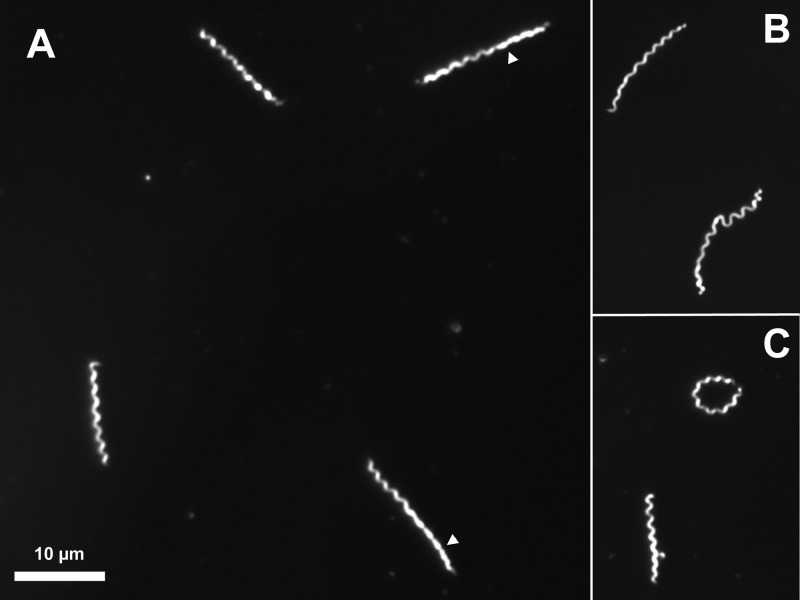
Appearance of *in vitro*-cultured T. pallidum subsp. pallidum Nichols by dark-field light microscopy. This specimen was from a 200-day culture from experiment 1. (A) Four organisms with typical morphology, showing regions with planar, “flat wave” morphology (arrowheads). (B) Example of the inherent flexibility of T. pallidum. (C) Ring-shaped organism in which the two ends are joined. All of these forms are also commonly seen in T. pallidum organisms freshly extracted from rabbit tissue.

T. pallidum exhibited characteristic morphology and vigorous motility during the course of *in vitro* culture, as examined by dark-field microscopy (Fig. 6). Regions of helical and planar wave morphology, as have been described previously for both T. pallidum (29) and Borrelia burgdorferi (30), were observed. Some organisms had circularized to form ring-shaped structures. Tangled clusters of 2 to 10 bacteria were also present. All of these forms are also commonly observed in T. pallidum extracted from infected rabbit tissue (not shown). Cell length was variable, reflecting a mixture of organisms in different stages of cell elongation and division.

**FIG 7:**
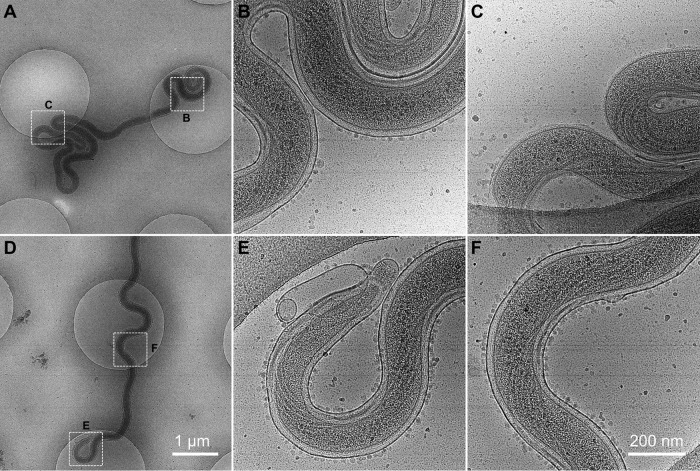
Structure of T. pallidum subsp. pallidum Nichols following 54 days of *in vitro* culture, as determined by cryo-electron microscopy. (A and D) Two T. pallidum cells were imaged at low magnification (bar, 1 µm). (B, C, E, and F) Highlighted areas in panels A and D were imaged at high magnification (bar, 200 nm). B, intact region in the middle of the cell, showing the binding of small (~10-nm) particles or vesicles to the outer membrane surface; C, region of the cell in panel A in which the outer membrane has been stripped off. Note the lack of bound particles. E, end of the cell in panel D, showing the conical tip structure and a prominent membrane bleb, both typical of T. pallidum structure; F, intact region in middle of cell. The outlines of periplasmic flagella are clearly visible in this and other panels.

Cryo-electron microscopy (Cryo-EM) was utilized to determine whether *in vitro*-cultured T. pallidum retained the characteristic structure of the organism. As shown in Fig. 7 and the cryo-electron tomography (cryo-ET) series in Movies S1 and S2 in the supplemental material, T. pallidum Nichols cultured for 52 days *in vitro* exhibited a structure that was indistinguishable from that of T. pallidum extracted from infected rabbit tissue (31). The outer membrane, periplasmic flagella, peptidoglycan layer, inner membrane, chemotaxis arrays, conical tip organelles, and cytoplasmic filaments are all clearly discernible and identical in appearance to those in rabbit-derived specimens. Interestingly, particles ~10 nm in diameter resembling small vesicles appeared to be bound to the outer membrane (Fig. 7). Figure 7C shows a region of an organism in which the fragile outer membrane has been stripped off, as commonly occurs in T. pallidum preparations. That region is devoid of the small particles, indicating that the particles are binding to the outer membrane surface.

10.1128/mBio.01153-18.8 MOVIE S1  T. pallidum subsp. pallidum Nichols cell from a 54-day *in vitro* culture visualized by cryo-electron tomography (cryo-ET). Each frame represents a slice in the *z* dimension of the cryo-ET 3D reconstruction. The cytoplasmic membrane, outer membrane (with membrane blebs), peptidoglycan layer, periplasmic flagella, cytoplasmic filaments, and external particles/vesicles bound to the outer membrane are clearly visible. Artifactual distortion of the cell at the edge of the hole in the carbon grid is present, consistent with the inherent flexibility of T. pallidum and other spirochetes. Download MOVIE S1, MOV file, 32.4 MB. Copyright © 2018 Edmondson et al. 2018 Edmondson et al. This content is distributed under the terms of the Creative Commons Attribution 4.0 International license.

10.1128/mBio.01153-18.9 MOVIE S2  Cryo-ET series for a second T. pallidum subsp. pallidum Nichols cell from a 54-day *in vitro* culture. This series is close to the tip of the cell and shows a portion of the conical tip structure and sections through at least three flagellar motors, as well as the features described in the legend of Movie S1. Download MOVIE S2, MOV file, 27.7 MB. Copyright © 2018 Edmondson et al. 2018 Edmondson et al. This content is distributed under the terms of the Creative Commons Attribution 4.0 International license.

### Parameters affecting *in vitro* growth of T. pallidum*.*

**FIG 8:**
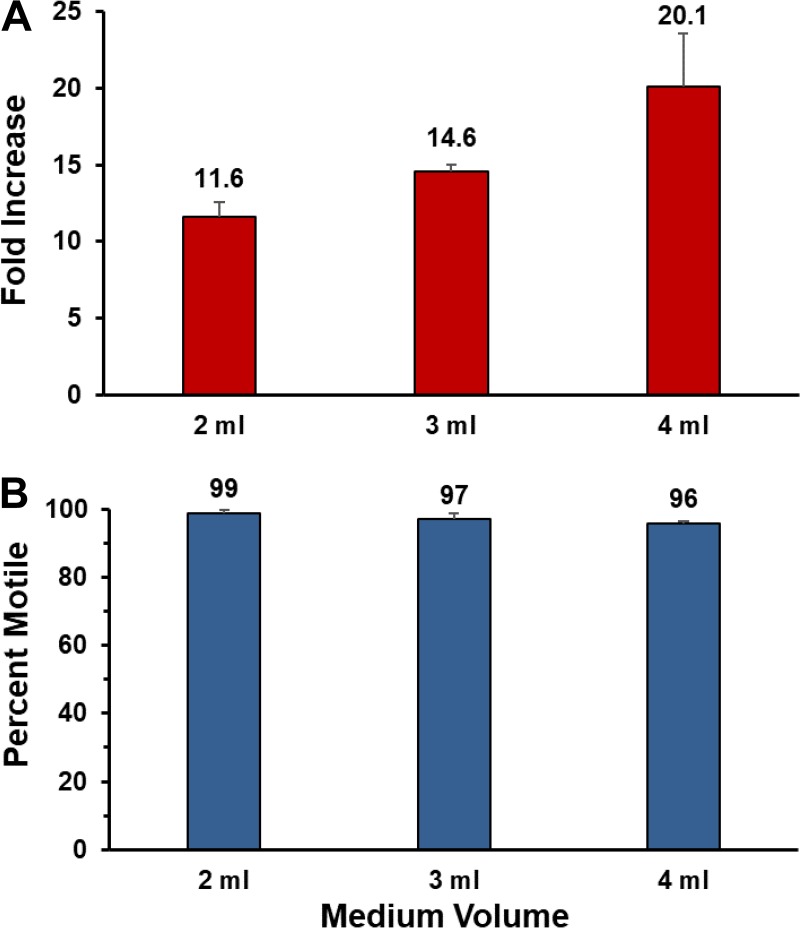
Effect of medium volume on T. pallidum multiplication in the Sf1Ep coculture system. Equal quantities of T. pallidum Nichols were inoculated into triplicate 9-cm^2^ Sf1Ep cell cocultures containing 2, 3, or 4 ml of TpCM-2. The cultures were harvested and evaluated for T. pallidum fold increase and motility on day 7. (A) Increased culture yield with higher medium volumes. (B) High retention of motility in all cultures.

We have begun the process of examining factors that affect T. pallidum multiplication in this *in vitro* system. It had been noted in previous studies that T. pallidum growth in primary cultures was limited when the concentration of bacteria was initially high (14–17). We reasoned that the availability of nutrients was limiting and therefore examined whether simply increasing the volume of TpCM-2 in the cultures could increase the yield and survival of T. pallidum. Increasing the volume of medium in 6-well cultures from 2 ml to 3 or 4 ml resulted in a concomitant increase in the yield per culture (Fig. 8). We therefore began to utilize 4 ml of TpCM-2 per culture in subsequent experiments.

The yield per culture could also be increased by enlarging the size of the cultures from the 6-well cluster dish format (9-cm^2^ surface area) to 75-cm^2^ tissue culture flasks. Each 75-cm^2^ flask has a 9.3-fold-higher surface area than a well in a 6-well cluster dish. A proportionately increased number of Sf1Ep cells, a higher medium volume (15 ml), and a larger inoculum were used, essentially “supersizing” the cultures. Yields were increased by roughly 7-fold to 10-fold in the 75-cm^2^ cultures in comparison with 6-well culture controls (see Table S1 in the supplemental material).

10.1128/mBio.01153-18.7 TABLE S1  Increased T. pallidum culture yield in 75-cm^2^ cultures relative to 9-cm^2^ 6-well cultures. Download TABLE S1, DOCX file, 0.01 MB. Copyright © 2018 Edmondson et al. 2018 Edmondson et al. This content is distributed under the terms of the Creative Commons Attribution 4.0 International license.

## DISCUSSION

Here, we report the first consistent long-term *in vitro* cultivation of T. pallidum subsp. pallidum. The successful culture of recent syphilis isolates UW231B and UW249B as well as the long-established Nichols strain indicates that the cultivation procedure will likely be applicable to other syphilis isolates. Given the extreme similarity of all the T. pallidum subspecies, T. carateum, and T. paraluiscuniculi, it is probable that the same conditions will be effective in propagating all the members of this group of pathogens, as will be examined in future studies.

The culture method used was strongly based on the prior studies by A. Howard Fieldsteel, David L. Cox, and their coworkers, who systematically established the requirements for growth of T. pallidum in primary cultures. The extended *in vitro* survival of T. pallidum in the presence of mammalian cells had been noted by several groups (32–37), and examination of several cell cultures indicated that Sf1Ep cells performed better than other cell types in this aspect (most likely because of their low growth rate and low metabolism). Fieldsteel et al. (37) used oxygen gradients formed in Leighton tubes to demonstrate that microaerobic conditions further prolonged survival and apparent multiplication. It was also noted that only certain commercial fetal bovine serum (FBS) lots supported T. pallidum viability (38). By combining these components with Eagle’s MEM and DTT to decrease the presence of reactive oxygen species, consistent *in vitro* multiplication was achieved and reproduced in many experiments. However, attempts to obtain continued growth through subculturing were generally unsuccessful, with the combined yields of the primary and passaged cultures rarely exceeding that of the primary culture alone. Cox obtained enhanced survival and growth with subculture in 4 experiments in which up to 2,000-fold increases over a 17-to-30-day period were observed (reviewed in reference 14). However, these results were not reproduced in subsequent experiments (14).

We believe that the key to achieving long-term culture of T. pallidum in the current study is the combination of the use of the TpCM-2 modified medium and the maintenance of near-homeostatic conditions through regular subculture and partial medium replacement as needed. TpCM-2 differs from its precursor, TpCM, by substitution of Eagle’s MEM with CMRL 1066 medium as the basal medium and the omission of some of the TpCM additives (Table 4). CMRL 1066 medium was selected because it is utilized as the basal medium in Barbour-Stoenner-Kelly (BSK) medium, which is widely used for culture of *Borrelia* (relapsing fever) and *Borreliella* (Lyme disease) isolates (39, 40). Compared to MEM, CMRL contains additional nutrients, including nucleic acid bases, nucleosides, cocarboxylase, coenzyme A, flavin adenine dinucleotide, NAD, NADP, Na acetate, Na glucuronate, and the fatty acid mixture Tween 80; some of the shared components are also present at different concentrations. Further analysis will be needed to determine (i) if the CMRL-MEM substitution is required for long-term T. pallidum culture and, if so, (ii) what CMRL component(s) is responsible for enhanced T. pallidum survival and growth. The other factor that appears to be important in prolonged survival and growth is regular subculture at 6-to-7-day intervals. The relatively short time between subcultures limits both the depletion of nutrients and the development of toxic conditions, e.g., changes in pH and accumulation of toxic by-products. It should be noted, however, that the subculture interval can be increased when the number of T. pallidum organisms in the culture is low and partial medium replacement is utilized. For example, in some of our low-inoculum experiments the level in the inocula became ≤10^6^ per culture. To bolster these cultures, they were at times extended by up to 15 days with partial medium replacement at 4-to-7-day intervals (see, e.g., Fig. 1, low inoculum, days 59 to 74).

**TABLE 4:** TpCM-2^a^

Component	Amt for 50 ml	Final concn	Manufacturer/catalog no.
1× CMRL 1066 medium without l-glutamine or phenol red	37 ml	0.8×	United States Biological/C5900-03A
Sodium pyruvate	364 µl	0.73 mM	Sigma/S8636
0.1% resazurin	50 µl	0.001%	Sigma/R7017
MOPS (1 M), pH 7.5	1 ml	20 mM	Sigma/M3183
NaHCO_3_ (7.5%)	1.08 ml	19.2 mM	Sigma/M8761
l-Glutamine (200 mM)	500 µl	2 mM	Sigma/G6152
100× d-glucose (15% in water)	500 µl	To 17.6 mM	Sigma/G6152
d-Mannitol (10 g/dl) (10% in water)	80 µl	0.88 mM	Sigma/M-1902
l-Histidine (5 g/dl) (5% in water)	80 µl	0.52 mM	Sigma/H6034
dl-Dithiothreitol	4 mg	0.52 mM	Sigma/D9779
Fetal bovine serum, heat inactivated	10 ml	20%	Sigma/F4135

a TpCM-2, T. pallidum cultivation medium 2; MOPS, morpholinepropanesulfonic acid.

Another observation was that a maximum yield of T. pallidum organisms per culture occurred in standard 9-cm^2^ cultures with 2 ml of TpCM-2; the yield per culture was limited despite inoculum size when all other conditions are held constant. We found that a simple increase in the volume of medium resulted in a proportionately improved yield when a high inoculum was used (Fig. 8). Therefore, it appears that T. pallidum itself consumes nutrients and/or alters culture conditions in a way that limits its *in vitro* growth, as is the case for most bacterial cultures. The yield of T. pallidum organisms per culture could also be increased by using 75-cm^2^ flasks and concomitant increases in inoculum size, medium volume, and Sf1Ep cell numbers (Table S1).

Optimal growth of T. pallidum in this system required the presence of Sf1Ep cells, with little multiplication occurring in parallel axenic cultures (Fig. 3; see also Fig. S3 in the supplemental material). It is of interest that the number of genome Equiv. per cell increased dramatically in axenic cultures, whereas this value remains relatively constant at ~3 genome Equiv. per cell in parallel cultures with Sf1Ep cells. Indeed, it had been observed previously that T. pallidum in an axenic environment continues to synthesize DNA (and RNA) for up to 6 days, despite the fact that the number of cells did not increase in these experiments (41, 42). Thus, the barrier to multiplication in the axenic cultures performed to date is not likely to be due to defects in genome replication. Direct interaction through adherence between Sf1Ep cells and T. pallidum is apparently required for promotion of treponemal multiplication in that separation of T. pallidum from the cell monolayer in Transwell chambers prevented growth and shortened survival (unpublished observations).

We speculate that T. pallidum may directly acquire certain nutrients, such as lipids, through direct interaction with host cells. It had been shown previously that B. burgdorferi can obtain lipids during direct interaction with mammalian cells (43) and that T. pallidum can acquire fluorescently labeled fatty acids directly from the surrounding medium (44). Matthews et al. (45) found that the lipid content of T. pallidum purified from infected rabbit testes had high proportions of cholesterol, which was most likely acquired directly from the host rather than synthesized from fatty acids. These are important observations, since neither of these organisms contains the genes required for fatty acid synthesis (46, 47). T. pallidum, like other treponemes (48–50), most likely acquires lipids bound to serum albumin, which acts as a detoxifying agent (51); perhaps this mechanism is supplemented by direct acquisition from host cells. The bound particles or vesicles observed by cryo-electron microscopy (Fig. 7; see also Movies S1 and S2 in the supplemental material) could conceivably represent a means of nutrient acquisition.

T. pallidum is a microaerophilic organism that requires low (1.5% to 5%) concentrations of oxygen for long-term survival and growth and yet is extremely sensitive to the toxic effects of atmospheric levels of oxygen (16, 36, 42, 52–55). In 1974, Cox and Barber (54) demonstrated that T. pallidum consumed O_2_, but even today we do not have a good explanation of how oxygen is utilized. T. pallidum lacks genes encoding the tricarboxylic cycle, cytochromes, or other components of typical bacterial oxidative phosphorylation pathways (47). One hypothesis is that O_2_ is used as an electron acceptor to maintain appropriate NADH/NAD^+^ levels through the action of NADH oxidase (56). T. pallidum lacks genes encoding superoxide dismutase or catalase but may utilize neelaredoxin to provide protective activity against superoxide and other reactive oxygen species (ROS) (57). Reducing compounds such as DTT in TpCM and TpCM-2 scavenge ROS and play an important role in prolonging T. pallidum survival *in vitro* (16). It is also possible that host cells *in vivo* (and Sf1Ep cells in the *in vitro* culture system) are active in scavenging of ROS and thus in protecting T. pallidum from these toxic compounds.

Sf1Ep cells are better at supporting the survival and growth of T. pallidum than are all other cell types that have been examined (58). The particular capability of Sf1Ep cells to support T. pallidum multiplication may be related to their relatively low growth rate and low metabolic activity. Sf1Ep cell cultures can survive quite well for 2 weeks, with little change in medium parameters such as pH. It is likely that other mammalian cell types deplete nutrients and change medium conditions more rapidly, limiting their efficacy in supporting T. pallidum growth. We are hopeful that future studies will identify the nutrients or protective activities provided by Sf1Ep cells and thus permit long-term axenic survival and growth.

Retention of infectivity in the rabbit model and structural integrity are important indicators that *in vitro*-cultured T. pallidum organisms maintain wild-type properties. In future analyses, the genome sequence of long-term-cultured treponemes will be compared with those of rabbit-propagated organisms to determine whether any sequence differences are evident. However, selection of a small subset of variant cells capable of growing *in vitro* seems implausible, because exponential multiplication is evident within days after the inoculation of cultures with rabbit-derived treponemes.

The members of the T. pallidum group of pathogens represent an extreme in terms of host dependence. These bacteria have adapted to survival only in mammalian tissue, relying on the host for provision of nucleic acid bases, fatty acids, most amino acids, and glucose as an energy source (14, 47, 56) as well as for the maintenance of near-homeostatic conditions in terms of temperature, osmolarity, oxygen and CO_2_ levels, and pH. The genus *Treponema* is a genetically diverse group whose known members are primarily host-associated organisms, including commensal skin organisms, oral treponemes, intestinal spirochetes, oral- and hoof-associated organisms involved in polymicrobial infections, and termite gut symbionts (59). However, T. caldaria, T. stenostreptum, and T. zuelzerae were recently recognized as free-living *Treponema* species (60); many more environmental species are likely, as indicated by the identification of multiple *Treponema* genomes in a microbiome study of water well sediment in Rifle, CO (61). Progressive genome reduction is extreme in this genus, resulting in decreases from roughly 4 Mb for environmental and termite-associated treponemes to only 1.1 Mb for the T. pallidum group, with intermediate genome sizes occurring in other mammal-associated *Treponema* species (18). Thus, T. pallidum has lost genes for most biosynthetic pathways, stress response pathways, and complex energy production pathways while retaining the minimum complement of genes required for survival, proliferation, and transmission in the near-homeostatic environment of mammalian tissue, including those encoding transporters and efficient motility and chemotaxis systems.

In summary, the modification of the system used by Fieldsteel et al. (15) described here will likely facilitate the characterization of the T. pallidum subspecies, T. carateum, and the rabbit and hare-infecting *Treponema* species. Future studies will include application of this approach to culture of T. pallidum subsp. pertenue and subsp. *endemicum*, which would in turn provide new avenues for the study of yaws and bejel. It is likely that the rabbit and hare pathogen (provisionally renamed “T. paraluisleporidarum” from T. paraluiscuniculi [62]) can also be cultured by this method, which may provide new insights into the evolution of host specificity among *Treponema* species. We anticipate that it will be possible to isolate T. pallidum and other pathogenic *Treponema* directly from tissues or body fluids using the *in vitro* culture system. Potentially, this approach also may permit the culture of the pinta organism T. carateum, which has been shown to be infectious in primates but has not been propagated in rabbits or other laboratory animals (19, 27). *In vitro*-cultured pathogenic treponemes could also be used in *in vitro* host-pathogen interaction and immunologic studies, as well as in vaccine development. We have already begun to utilize the culture system for the following purposes: delineation of the nutrients required for *in vitro* growth; comparison of the transcriptome and proteome of *in vitro*-cultured T. pallidum with those of rabbit-propagated organisms; cloning of pathogenic *Treponema* by limiting dilution; genome sequencing of *in vitro*-cultured T. pallidum; and antimicrobial susceptibility testing. Random and targeted mutagenesis studies may also be possible using the T. pallidum culture system. However, the current procedure is still complex, due to the requirement for tissue culture cells and a microaerobic environment. This aspect may hinder its widespread use by research laboratories, as well as its potential application to clinical uses. Therefore, the primary goal remains the development of an axenic system that supports the long-term culture of pathogenic *Treponema* species, thereby further simplifying the study of these enigmatic organisms.

## MATERIALS AND METHODS

### Ethics statement.

All procedures involving rabbits were reviewed and approved by the Animal Welfare Committee of the University of Texas Health Science Center at Houston.

### Tissue culture.

All reagents were purchased from Sigma-Aldrich unless otherwise indicated. Sf1Ep (NBL-11) cells (ATCC CCL-68) were obtained from the American Type Culture Collection, Rockville, MD. Stocks of Sf1EP cells were between passage 19 and 40 and were maintained in Sf1Ep medium consisting of Eagle’s MEM with nonessential amino acids, l-glutamine, sodium pyruvate, and 10% heat-inactivated FBS (51) at 37°C in air with 5% CO_2_. Low-passage Sf1Ep cells grow slowly and were subcultured at 1:5 every 2 to 3 weeks. High-passage cells grow more rapidly and were subcultured at 1:20 every week. Cells were fed weekly by replacement of one-half of the medium volume.

### Bacteria.

Treponema pallidum subspecies pallidum Nichols, initially isolated from the cerebrospinal fluid of a neurosyphilis patient in Baltimore, MD, in 1912 (20), was obtained from J. N. Miller at the UCLA Geffen School of Medicine. The UW231B and UW249B strains were isolated in 2004 from the blood of untreated syphilis patients in Seattle, WA, and were the kind gift of L. C. Tantalo, S. K. Sahi, and C. M. Marra (University of Washington [UW] School of Medicine). T. pallidum strains were maintained by intratesticular passage in male New Zealand White rabbits (3 to 4 kg) that were housed at 16 to 18°C and were provided antibiotic-free food and water. Rabbits were inoculated with 2 × 10^7^ to 5 × 10^7^ organisms from frozen stocks per testis. Animals infected with the Nichols strain were euthanized at the time of peak orchitis (10 to 12 days). Infection with the UW strains was monitored by rapid plasma reagin (RPR) test reactivity, and the animals were euthanized when a positive test result was obtained (20 to 40 days for UW231B, 26 days for UW249B). Testes were removed aseptically, and T. pallidum organisms were extracted from minced testes by gentle stirring in 10 ml of filter-sterilized phosphate-buffered saline (PBS; 0.154 M NaCl, 0.01 M NaHPO_4_, pH 7.4) with 50% heat-inactivated rabbit serum and 1 mM DTT for 10 min. Rabbit cells were removed by centrifugation at 100 × *g* for 7 min. The T. pallidum suspension was supplemented with 15% (vol/vol) sterile glycerol and stored in 1-ml aliquots at −80°C. *In vitro*-cultured T. pallidum was frozen in TpCM-2 medium supplemented with 15% glycerol.

### T. pallidum cultivation medium.

TpCM-2 was prepared as indicated in Table 4 1 day prior to use. All solutions were made in cell culture-grade water (Sigma W3500) and filter sterilized. Fetal bovine serum (FBS) lots vary considerably in their ability to support T. pallidum survival and replication (16, 38, 51). FBS lots from several suppliers were therefore prescreened for efficacy (by comparison with previously tested lots), purchased in bulk, and heat inactivated at 56°C for 30 min prior to use. Samples of FBS lots that support the multiplication of T. pallidum are available upon request. The pH of the medium was adjusted to 7.5, and the mixture was then filter sterilized with 0.22-µm-pore-size polyethersulfone filters (Merck Millipore). The medium was then preequilibrated in a BBL GasPak jar in which a vacuum was drawn five times (house vacuum; ~12 to 18 µm Hg), and the jar was refilled with 5% CO_2_–95% N_2_ four times and a final time with 1.5% O_2_–5% CO_2_–93.5% N_2_. The medium was then incubated overnight in a Forma model 3130 trigas incubator (Thermo Fisher) maintained at 34°C with 1.5% O_2_–5% CO_2_–93.5% N_2_ (here referred to as the low-oxygen incubator). All subsequent steps in the incubation of T. pallidum cultures were carried out under these conditions.

### *In vitro* cultivation of T. pallidum.

At 1 to 2 days prior to each culture initiation or passage, Sf1Ep cells were trypsinized and seeded in tissue culture-treated 6-well cluster plates (Falcon 353046) at 0.5 to 1 × 10^5^ cells per well. Due to the long culture period, the use of plates with low-evaporation lids is essential. At least 3 h prior to the start of an experiment or passage, the medium in the 6-well plates containing Sf1EP cells was removed, and the plates were rinsed with the preequilibrated TpCM-2 to remove any traces of Sf1Ep medium prior to addition of 2 to 4 ml of the TpCM-2. Plates were then preequilibrated in the GasPak jar as described above and transferred to the low-oxygen incubator. All manipulations of cultures were carried out in ambient air with use of a laminar flow hood, with efforts to limit this air exposure to <30 min to limit oxygen exposure.

Cultivation experiments were initiated using frozen aliquots of T. pallidum extracted from rabbits. A frozen aliquot was thawed at room temperature and diluted in TpCM-2, such that each 50-to-100-µl inoculum contained 0.5 × 10^6^ to 1.25 × 10^6^ T. pallidum. Sf1Ep cultures were briefly removed from the incubator, inoculated with T. pallidum, preequilibrated, and then returned to the low-oxygen incubator.

### Subculture procedure.

After 6 to 7 days of incubation, T. pallidum organisms were subcultured as follows. The TpCM-2 medium was removed and reserved. Each well was rinsed with 0.35 ml of trypsin-EDTA (Sigma T4049), which was combined with the reserved medium. An additional 0.35 ml of trypsin-EDTA was added, and the plates were incubated at 37°C for 5 min. The reserved medium, the rinsed and trypsinized Sf1Ep cells, and the T. pallidum organisms were combined and used to rinse the culture well. An aliquot (ranging from 100 to 500 µl) of the trypsinized culture was used to inoculate new 6-well Sf1Ep cultures prepared as described above. A minimum of three wells were inoculated per condition.

### Quantification of T. pallidum.

A minimum of three biological replicates were used for each condition and time point. Trypsinized cultures were quantitated by dark-field microscopy using one of two counting methods. Samples (10 µl) were placed under 22-by-22-mm coverslips on plain glass slides, and the numbers of T. pallidum organisms in random fields were counted using a 40× objective lens until 10 fields or >50 organisms had been counted. The motility of each organism was also assessed. At least three counts were made for each sample. Using calibration of the field diameter with a stage micrometer, the concentration of organisms per milliliter was estimated as the average number per field ×10^6^/2.2. Alternatively, cultures were quantitated using Helber counting chambers with Thoma rulings (Hawksley, Lancing, Sussex, United Kingdom). Each culture was counted at least twice using this method. Data for ongoing experiment 2 are those collected through 10 May 2018, whereas data from ongoing experiments 1, 3, and 4 are those collected prior to 28 April 2018.

### Quantitative PCR.

Trypsinized cultures were centrifuged at 14,000 × *g* for 10 min to pellet T. pallidum. DNA from the pelleted organisms was purified using a DNeasy kit (Qiagen), and 1/50 of the material (2 µl of a total of 100 µl) was used for each of three quantitative PCR (qPCR) reactions per sample. qPCR was performed using iQ Sybr green supermix on a C1000 Touch Thermal Cycler (Bio-Rad). The primers that were targeted to the T. pallidum DNA polymerase I gene (*polA*; TPANIC_0105) were as follows: 6037TP1 (5′-CAGGATCCGGCATATGTCC-3′) and 6037TP2 (5′-AAGTGTGAGCGTCTCATCATTCC-3′). The program consisted of 95°C for 2 min followed by 39 cycles of 95°C for 5 s and 60°C for 30 s. All samples were examined in triplicate technical replicates, and standard curves using purified T. pallidum DNA were performed for each plate and had linear regression coefficients of determination (*R*^2^) of ≥0.98. No-template controls (NTC) and DNA extracted from uninfected Sf1Ep cultures were used as controls to optimize the qPCR assay conditions.

### Infectivity studies.

The infectivity of cultured T. pallidum was determined by injecting serial dilutions of the sample intradermally into the shaved backs of rabbits. Dilutions were performed in TpCM-2, and each dilution was inoculated at duplicate sites on each rabbit. The inoculation sites were shaved and examined daily for 45 days for the development of the occurrence of erythema and induration, which together constitute lesion development. Needle aspirates of representative lesions (two per dosage) were examined by dark-field microscopy for the presence of motile treponemes indicating active treponemal infection. Rabbits were provided antibiotic-free food and water, housed at 16 to 18°C, and shaved daily throughout the course of the experiment. Median 50% infectious dose (ID_50_) values were determined by the method of Reed and Muench (63).

### Light micrography.

Digital photographs were obtained as described previously (64) using a Nikon Eclipse microscope (Nikon, Tokyo, Japan) equipped with Cytoviva X-cite 120 dark-field illumination (Cytoviva, Auburn, AL), a CoolSNAP HQ charge-coupled-device (CCD) camera (Photometrics, Tucson, AZ), and Nikon NIS-Elements AR3.2 software. Photography was performed with a 100× objective lens with an internal diaphragm (Nikon) using oil immersion.

### Cryo-electron microscopy.

Bacterial cultures were mixed with 10-nm-diameter gold particle clusters, which were used as fiducial markers, and were then deposited onto freshly glow-discharged, holey carbon grids for 1 min. The grids were blotted with filter paper and rapidly frozen in liquid ethane, using a gravity-driven plunger apparatus. The frozen-hydrated specimens were imaged at −170°C using a Polara G2 electron microscope (FEI Company) equipped with a field emission gun and a direct-detection device (Gatan K2 Summit). The microscope was operated at 300 kV with a magnification of ×9,400, resulting in an effective pixel size of 4.5 Å at the specimen level. We used SerialEM (65) to collect low-dose, single-axis tilt series with the dose fractionation mode at about 6-µm defocus and a cumulative dose of ~60 e^−^/Å^2^ distributed over 35 stacks. Each stack contains ~8 images. Tilt series were collected at angles from −51° to 51° with increments of 3°. We used Tomoauto (66) to facilitate data processing, which included drift correction of dose-fractionated data using Motioncorr (67) and assembly of corrected sums into tilt series, automatic fiducial seed model generation, alignment and contrast transfer function correction of tilt series by IMOD (68), and reconstruction of tilt series into tomograms by Tomo3D (69).
