# Supplementary figures and images for: Long-Term In Vitro Culture of the Syphilis Spirochete Treponema pallidum subsp. pallidum
Source: mBio. 2018 Jun 26;9(3):e01153-18. doi: 10.1128/mBio.01153-18 (PMC6020297; doi:10.1128/mBio.01153-18)

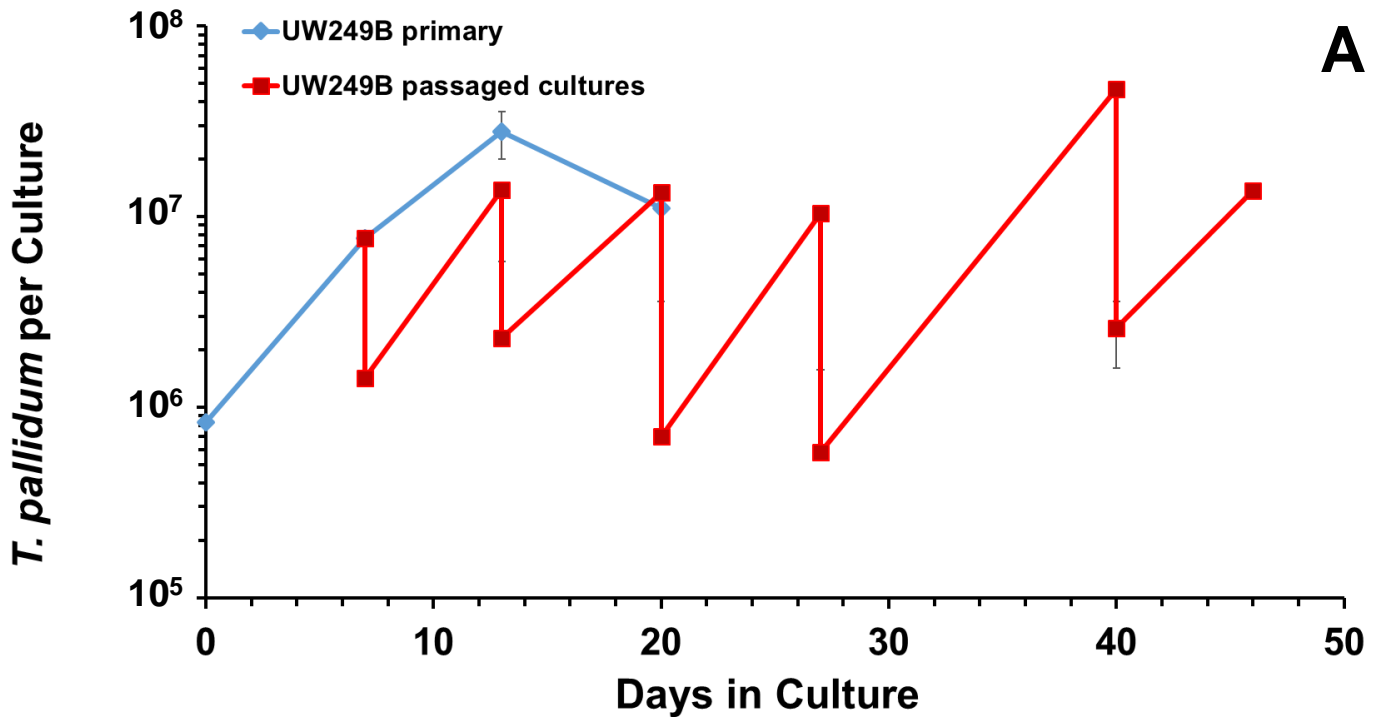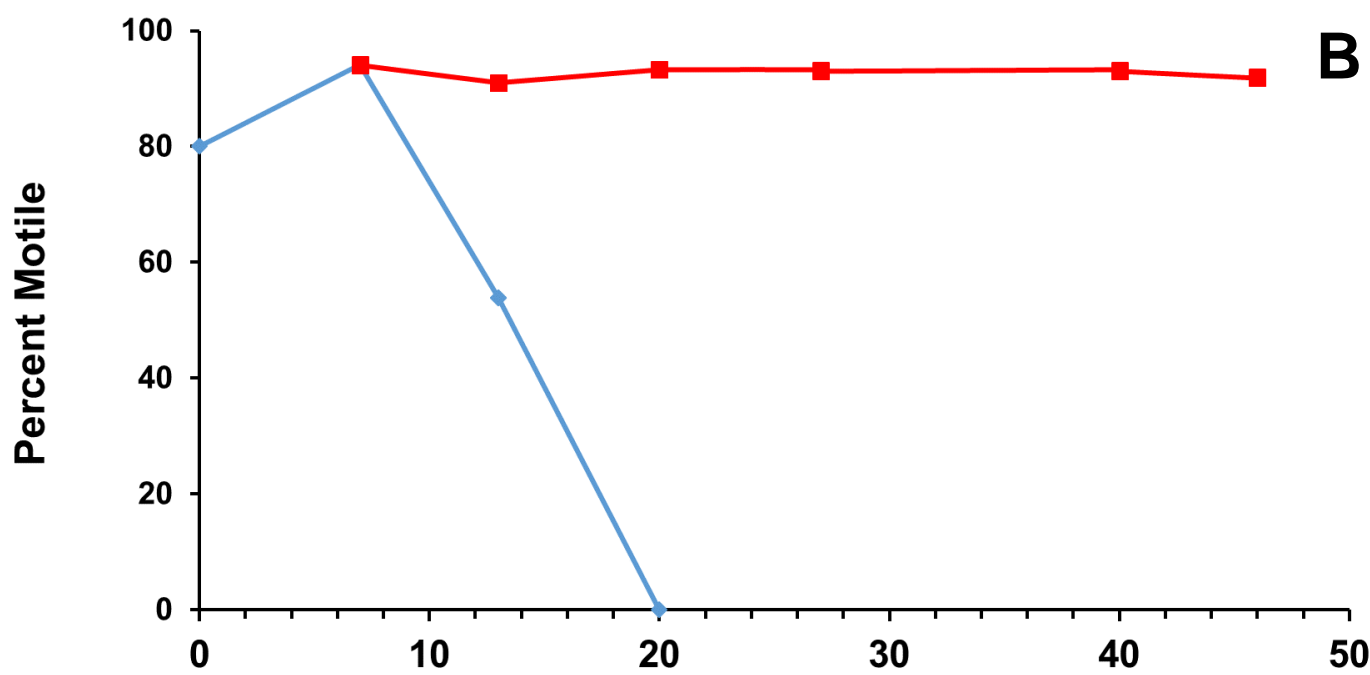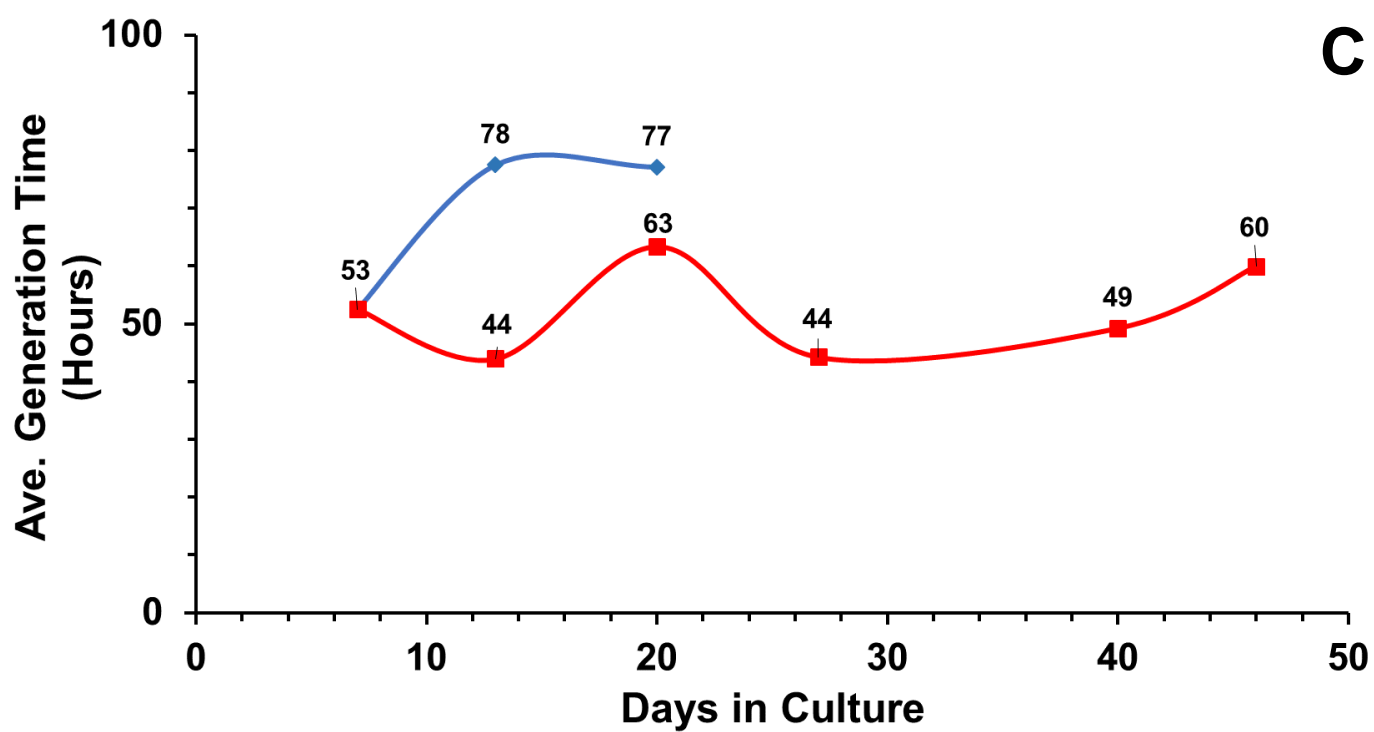

Supplement: FIG S6 [file mbo003183947sf6.pdf]
