# Supplementary material for: Long-Term In Vitro Culture of the Syphilis Spirochete Treponema pallidum subsp. pallidum
Source: mBio. 2018 Jun 26;9(3):e01153-18. doi: 10.1128/mBio.01153-18 (PMC6020297; doi:10.1128/mBio.01153-18)
Supplement: TABLE S1 [file mbo003183947st1.docx]

| Table S1. Increased *T. pallidum* culture yield in 75 cm^2^ cultures relative to 9 cm^2^ 6-well cultures^a^ | | | | | | | |
| --- | --- | --- | --- | --- | --- | --- | --- |
| **Experiment** | **6-well medium**  **volume** | **6-well yield**  **(motility)** | **6-well fold increase** | **75 cm^2^ flask medium**  **volume** | **75 cm^2^ flask yield (motility)** | **75 cm^2^ flask**  **fold increase** | **Yield ratio^b^** |
| 1 | 2 | 4.5 x 10^7^  (99%) | 12 | 15 | 3.9 x 10^8^  (97%) | 9 | 8.7 |
| 2 | 4 | 8.6 x 10^7^  (98%) | 21 | 15 | 8.7 x10^8^  (97%) | 23 | 10.0 |
| 3 | 4 | 6.5 x 10^7^  (91%) | 14 | 15 | 4.7 x 10^8^  (97%) | 13 | 7.2 |
| 4 | 4 | 4.2 x 10^7^  (99%) | 30 | 15 | 3.0 x 10^8^  (98%) | 24 | 7.3 |
| 5 | 4 | 9.2 x 10^7^  (97%) | 21 | 15 | 9.5 x 10^8^  (99%) | 25 | 10.3 |

^a^ Cultures were inoculated with in vitro-derived *T. pallidum* Nichols. The number of organisms added to the 75 cm^2^ flasks was 9.3X more than added to the 6-well cultures, in proportion to the relative surface area of the cultures. Cultures were incubated for 7 days under standard conditions as described in the Materials and Methods.

^b^ Yield ratio = (75 cm^2^ flask yield)/(6-well culture yield)
